# 2-Amino-6-(pyrrolidin-1-yl)-4-*p*-tolyl­pyridine-3,5-dicarbonitrile

**DOI:** 10.1107/S1600536811026092

**Published:** 2011-07-09

**Authors:** S. Antony Inglebert, Jayabal Kamalraja, Gnanasambandam Vasuki, K. Sethusankar

**Affiliations:** a Physics Department, Sri Ram Engineering College, Chennai 602 024, India; b Department of Chemistry, Pondichery University, Pondichery 605 014, India; c Department of Physics, RKM Vivekananda College (Autonomous), Chennai 600 004, India

## Abstract

In the title compound, C_18_H_17_N_5_, the pyrrolidine ring adopts an envelope conformation. The pyrrolidine ring is disordered over two sets of sites with occupancy factors of 0.648 (6) and 0.352 (6). The dihedral angles between the pyrrolidine and pyridine rings are 14.6 (3)° for the major component and 16.2 (6)° for the ninor component. The crystal structure is stabilized by inter­molecular N—H⋯N and C—H⋯N inter­actions.

## Related literature

For a related structure, see: Wang *et al.* (2011 ▶). For the biological activity of spiro compounds, see: Kobayashi *et al.* (1991 ▶); James *et al.* (1991 ▶). For the use of 2-amino-3-cyano­pyridines as inter­mediates in the preparation of heterocyclic compounds, see: Shishoo *et al.* (1983 ▶). For puckering parameters, see: Cremer & Pople (1975 ▶).
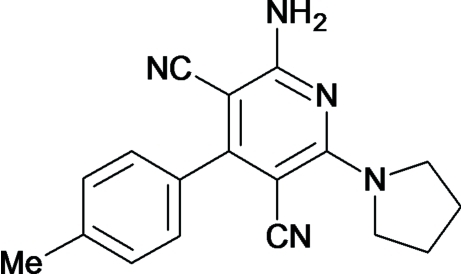


## Experimental

### 

#### Crystal data

C_18_H_17_N_5_
*M* *_r_* = 303.37
Triclinic, 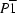

*a* = 7.4005 (5) Å
*b* = 9.0330 (5) Å
*c* = 12.0533 (6) Å
α = 87.876 (5)°
β = 80.575 (5)°
γ = 84.053 (5)°
*V* = 790.43 (8) Å^3^
*Z* = 2
Mo *K*α radiation
μ = 0.08 mm^−1^
*T* = 295 K
0.30 × 0.25 × 0.20 mm

#### Data collection

Bruker Kappa APEXII CCD diffractometer
Absorption correction: multi-scan (*SADABS*; Sheldrick, 1996 ▶) *T*_min_ = 0.916, *T*_max_ = 0.984
5515 measured reflections
2924 independent reflections
1812 reflections with *I* > 2σ(*I*)
*R*_int_ = 0.026

#### Refinement

*R*[*F* ^2^ > 2σ(*F* ^2^)] = 0.044
*wR*(*F* ^2^) = 0.119
*S* = 0.95
2924 reflections
222 parameters
48 restraints
H-atom parameters constrained
Δρ_max_ = 0.20 e Å^−3^
Δρ_min_ = −0.22 e Å^−3^

### 

Data collection: *APEX2* (Bruker, 2008) ▶; cell refinement: *SAINT* (Bruker, 2008) ▶; data reduction: *SAINT* ▶; program(s) used to solve structure: *SHELXS97* (Sheldrick, 2008 ▶); program(s) used to refine structure: *SHELXL97* (Sheldrick, 2008 ▶); molecular graphics: *ORTEP-3* (Farrugia, 1997 ▶); software used to prepare material for publication: *SHELXL97* and *PLATON* (Spek, 2009) ▶.

## Supplementary Material

Crystal structure: contains datablock(s) global, I. DOI: 10.1107/S1600536811026092/rk2281sup1.cif

Structure factors: contains datablock(s) I. DOI: 10.1107/S1600536811026092/rk2281Isup2.hkl

Supplementary material file. DOI: 10.1107/S1600536811026092/rk2281Isup3.cml

Additional supplementary materials:  crystallographic information; 3D view; checkCIF report

## Figures and Tables

**Table 1:** Hydrogen-bond geometry (Å, °)

*D*—H⋯*A*	*D*—H	H⋯*A*	*D*⋯*A*	*D*—H⋯*A*
N1—H1*A*⋯N4^i^	0.86	2.19	3.010 (2)	160
C11—H11⋯N2^ii^	0.93	2.62	3.531 (2)	166

Symmetry codes: (i) 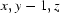; (ii) 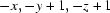.

**Fig. 1.**
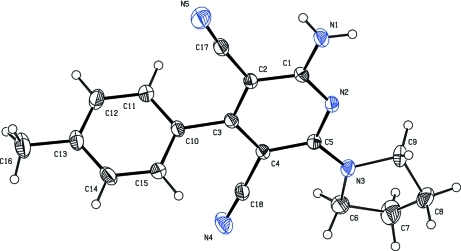
The molecular structure of the title compound with the atom numbering scheme. Displacement ellipsoids are drawn at 30% probability level. H atoms are present as small spheres of arbitary radius. Only major moiety of pyrrolidine is presented for clarity.

**Fig. 2.**
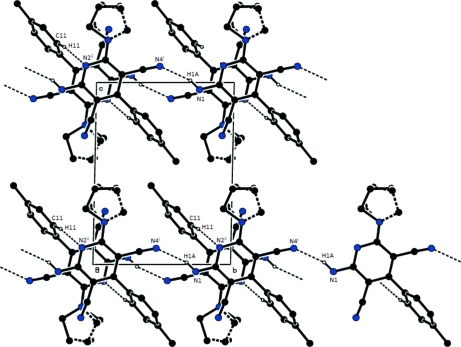
The packing arrangement of the title compound viewed down. Dashed lines indicates the N—H···N and C—H···N interactions.

## supplementary crystallographic information

### Comment

Generally the 'spiro'–compounds are naturally occurring substances (Kobayashi *et al.*, 1991; James *et al.*, 1991). Pyridines are of interest because of occurrence of their saturated and partially saturated derivatives in biologically active compounds and natural products such as *NAD* nucleotides, pyridoxol and pyridine alkaloids. Derivatives of 2-amino-3-cynaopyridine are important compound in the preparation of various hetrocyclic compounds (Shishoo *et al.*, 1983).

The title compound C_18_H_17_N_5_ was prepared from 4–methylbenzaldehyde, malononitrile and pyrrolidine. The reported compound pyridine bearing a pyrrolidine ring at C5 and benzene ring at C3. The X–ray analysis confirms the molecular structure and atom connectivity of the compound, as illustrated in Fig. 1. The pyrrolidine ring adopts an envelope conformation with puckering parameter q_2_ = 0.333 (8)Å, and φ_2_ = 98.1 (14)°, (Cremer & Pople, 1975) and the maximum deviation of C8 atom is -0.207 (6)Å. Also it has disordered with the occupancy factor of 0.648 (6) / 0.352 (6).

The pyridine ring (N2/C1—C5) forms dihedral angles of 14.6 (3)° and 59.89 (8)° with pyrrolidine (N3/C6—C9) and phenyl ring (C10—C15) respectively. Also the pyrrolidine ring forms a dihedral angle of 73.2 (3)° with phenyl ring. The title compound exibits the structural similarities with the reported related structure (Wang *et al.*, 2011).

The crystal structure is stabilized by N—H···N and C—H···N intermolecular interactions (Table 1). For the symmetry codes, see Table 1 too. The packing view of the reported compound is shown in Fig. 2.

### Experimental

Initially a mixture of 4–methylbenzaldehyde (2 mmoL,0.24 g), malononitrile (3 mmoL, 0.198 g), pyrrolidine (1.5 mmoL, 0.1 g) and was stirred without any solvent at room temperature. A solid appeared immediately which has dissolved in a minimum amount (3 ml) of ethanol and the solution was refluxed until completion of the reaction (monitered by *TLC*). The reaction mixture was cooled. Ethanol was evaporated under reduced pressure and the residue was extracted with dicholoromethane (3 x 10 ml). Evaporation of solvent left the crude solid which was subjected to silica gel column chromatography [25%/75% ethyl acetate/hexane] and the product was recrysallized from dichloromethane.

### Refinement

The H atoms were placed in idealized positions and allowed to ride on the parent atoms, with C—H bond lengths fixed to 0.93Å (Aromatic H), 0.96Å (methyl H), 0.97Å (methylene H), 0.86Å (N—H) and *U*_iso_(H) = 1.2–1.5*U*_eq_(C,*N*).

### Crystal data

**Table:**

C_18_H_17_N_5_	*Z* = 2
*M**_r_* = 303.37	*F*(000) = 320
Triclinic, *P*1	*D*_x_ = 1.275 Mg m^−^^3^
Hall symbol: -P 1	Mo *K*α radiation, λ = 0.71073 Å
*a* = 7.4005 (5) Å	Cell parameters from 2784 reflections
*b* = 9.0330 (5) Å	θ = 2.8–25.5°
*c* = 12.0533 (6) Å	µ = 0.08 mm^−^^1^
α = 87.876 (5)°	*T* = 295 K
β = 80.575 (5)°	Block, colourless
γ = 84.053 (5)°	0.30 × 0.25 × 0.20 mm
*V* = 790.43 (8) Å^3^	

### Data collection

**Table:**

Bruker Kappa APEXII CCD diffractometer	2924 independent reflections
Radiation source: fine–focus sealed tube	1812 reflections with *I* > 2σ(*I*)
graphite	*R*_int_ = 0.026
ω and φ scans	θ_max_ = 25.5°, θ_min_ = 2.8°
Absorption correction: multi-scan (*SADABS*; Sheldrick, 1996)	*h* = −8→8
*T*_min_ = 0.916, *T*_max_ = 0.984	*k* = −10→6
5515 measured reflections	*l* = −14→14

### Refinement

**Table:**

Refinement on *F*^2^	Primary atom site location: structure-invariant direct methods
Least-squares matrix: full	Secondary atom site location: difference Fourier map
*R*[*F*^2^ > 2σ(*F*^2^)] = 0.044	Hydrogen site location: inferred from neighbouring sites
*wR*(*F*^2^) = 0.119	H-atom parameters constrained
*S* = 0.95	*w* = 1/[σ^2^(*F*_o_^2^) + (0.0686*P*)^2^] where *P* = (*F*_o_^2^ + 2*F*_c_^2^)/3
2924 reflections	(Δ/σ)_max_ < 0.001
222 parameters	Δρ_max_ = 0.20 e Å^−^^3^
48 restraints	Δρ_min_ = −0.22 e Å^−^^3^

### Special details

**Table:**

Geometry. All s.u.'s (except the s.u. in the dihedral angle between two l.s. planes) are estimated using the full covariance matrix. The cell s.u.'s are taken into account individually in the estimation of s.u.'s in distances, angles and torsion angles; correlations between s.u.'s in cell parameters are only used when they are defined by crystal symmetry. An approximate (isotropic) treatment of cell s.u.'s is used for estimating s.u.'s involving l.s. planes.
Refinement. Refinement of *F*^2^ against ALL reflections. The weighted *R*–factor *wR* and goodness of fit *S* are based on *F*^2^, conventional *R*–factors *R* are based on *F*, with *F* set to zero for negative *F*^2^. The threshold expression of *F*^2^ > σ(*F*^2^) is used only for calculating *R*–factors(gt) *etc*. and is not relevant to the choice of reflections for refinement. *R*–factors based on *F*^2^ are statistically about twice as large as those based on *F*, and *R*–factors based on ALL data will be even larger.

### Fractional atomic coordinates and isotropic or equivalent isotropic displacement parameters (Å^2^)

**Table:**

	*x*	*y*	*z*	*U*_iso_*/*U*_eq_	Occ. (<1)
C1	0.2478 (2)	0.39231 (19)	0.48703 (14)	0.0311 (4)	
C2	0.2350 (2)	0.50607 (19)	0.40420 (14)	0.0299 (4)	
C3	0.2438 (2)	0.65256 (19)	0.43307 (14)	0.0287 (4)	
C4	0.2583 (2)	0.68285 (19)	0.54390 (14)	0.0295 (4)	
C5	0.2649 (2)	0.56120 (19)	0.62372 (14)	0.0293 (4)	
C6	0.2432 (9)	0.7131 (12)	0.7968 (9)	0.0461 (15)	0.648 (6)
H6A	0.1327	0.7729	0.7820	0.055*	0.648 (6)
H6B	0.3472	0.7716	0.7782	0.055*	0.648 (6)
C7	0.2226 (9)	0.6588 (7)	0.9190 (4)	0.0691 (15)	0.648 (6)
H7A	0.2771	0.7236	0.9638	0.083*	0.648 (6)
H7B	0.0939	0.6557	0.9508	0.083*	0.648 (6)
C8	0.3238 (8)	0.5040 (6)	0.9150 (3)	0.0563 (11)	0.648 (6)
H8A	0.2748	0.4437	0.9790	0.068*	0.648 (6)
H8B	0.4542	0.5084	0.9151	0.068*	0.648 (6)
C9	0.2914 (10)	0.4408 (11)	0.8058 (7)	0.0390 (12)	0.648 (6)
H9A	0.3947	0.3718	0.7741	0.047*	0.648 (6)
H9B	0.1800	0.3905	0.8169	0.047*	0.648 (6)
C6'	0.301 (2)	0.715 (3)	0.7863 (18)	0.0461 (15)	0.352 (6)
H6'1	0.1965	0.7891	0.7859	0.055*	0.352 (6)
H6'2	0.4114	0.7569	0.7482	0.055*	0.352 (6)
C7'	0.3223 (17)	0.6648 (14)	0.9056 (9)	0.0691 (15)	0.352 (6)
H7'1	0.4510	0.6416	0.9124	0.083*	0.352 (6)
H7'2	0.2680	0.7410	0.9590	0.083*	0.352 (6)
C8'	0.2208 (14)	0.5274 (12)	0.9249 (7)	0.0563 (11)	0.352 (6)
H8'1	0.2725	0.4600	0.9787	0.068*	0.352 (6)
H8'2	0.0914	0.5536	0.9531	0.068*	0.352 (6)
C9'	0.243 (2)	0.463 (2)	0.8208 (15)	0.0390 (12)	0.352 (6)
H9'1	0.1343	0.4147	0.8136	0.047*	0.352 (6)
H9'2	0.3477	0.3873	0.8132	0.047*	0.352 (6)
C10	0.2397 (2)	0.77241 (18)	0.34444 (14)	0.0311 (4)	
C11	0.0942 (3)	0.7948 (2)	0.28509 (15)	0.0401 (5)	
H11	−0.0063	0.7399	0.3045	0.048*	
C12	0.0959 (3)	0.8974 (2)	0.19760 (16)	0.0475 (5)	
H12	−0.0041	0.9107	0.1593	0.057*	
C13	0.2424 (3)	0.9812 (2)	0.16525 (15)	0.0416 (5)	
C14	0.3849 (3)	0.9602 (2)	0.22687 (16)	0.0478 (5)	
H14	0.4842	1.0165	0.2082	0.057*	
C15	0.3855 (3)	0.8584 (2)	0.31535 (15)	0.0395 (5)	
H15	0.4836	0.8477	0.3553	0.047*	
C16	0.2469 (4)	1.0884 (2)	0.06669 (17)	0.0647 (7)	
H16A	0.3279	1.1626	0.0743	0.097*	
H16B	0.1252	1.1356	0.0645	0.097*	
H16C	0.2907	1.0354	−0.0016	0.097*	
C17	0.2191 (3)	0.4648 (2)	0.29346 (16)	0.0400 (5)	
C18	0.2644 (3)	0.8343 (2)	0.57125 (14)	0.0367 (5)	
N1	0.2465 (2)	0.24897 (16)	0.46085 (13)	0.0465 (5)	
H1A	0.2550	0.1801	0.5115	0.056*	
H1B	0.2372	0.2262	0.3935	0.056*	
N2	0.26256 (19)	0.41952 (15)	0.59258 (11)	0.0333 (4)	
N3	0.2732 (2)	0.57631 (16)	0.73317 (12)	0.0370 (4)	
N4	0.2699 (3)	0.95712 (19)	0.58907 (14)	0.0591 (5)	
N5	0.2055 (3)	0.4201 (2)	0.20790 (15)	0.0654 (6)	

### Atomic displacement parameters (Å^2^)

**Table:**

	*U*^11^	*U*^22^	*U*^33^	*U*^12^	*U*^13^	*U*^23^
C1	0.0345 (10)	0.0243 (10)	0.0349 (11)	−0.0038 (8)	−0.0068 (8)	0.0012 (8)
C2	0.0353 (10)	0.0275 (10)	0.0272 (9)	−0.0050 (8)	−0.0057 (7)	0.0028 (8)
C3	0.0287 (9)	0.0263 (10)	0.0308 (9)	−0.0035 (8)	−0.0043 (7)	0.0024 (8)
C4	0.0347 (10)	0.0244 (9)	0.0300 (10)	−0.0044 (8)	−0.0061 (8)	0.0015 (8)
C5	0.0296 (10)	0.0298 (10)	0.0292 (10)	−0.0041 (8)	−0.0066 (8)	0.0027 (8)
C6	0.065 (4)	0.0426 (13)	0.032 (2)	−0.011 (4)	−0.006 (3)	−0.0053 (14)
C7	0.109 (4)	0.066 (2)	0.0320 (18)	−0.008 (4)	−0.009 (3)	−0.0056 (15)
C8	0.063 (3)	0.076 (2)	0.0343 (15)	−0.017 (3)	−0.016 (2)	0.0116 (15)
C9	0.048 (4)	0.037 (3)	0.027 (3)	0.010 (3)	−0.001 (2)	0.0050 (19)
C6'	0.065 (4)	0.0426 (13)	0.032 (2)	−0.011 (4)	−0.006 (3)	−0.0053 (14)
C7'	0.109 (4)	0.066 (2)	0.0320 (18)	−0.008 (4)	−0.009 (3)	−0.0056 (15)
C8'	0.063 (3)	0.076 (2)	0.0343 (15)	−0.017 (3)	−0.016 (2)	0.0116 (15)
C9'	0.048 (4)	0.037 (3)	0.027 (3)	0.010 (3)	−0.001 (2)	0.0050 (19)
C10	0.0423 (11)	0.0238 (10)	0.0267 (9)	−0.0039 (8)	−0.0047 (8)	0.0028 (8)
C11	0.0449 (12)	0.0404 (12)	0.0372 (11)	−0.0111 (9)	−0.0106 (9)	0.0078 (9)
C12	0.0590 (13)	0.0473 (13)	0.0381 (11)	−0.0014 (11)	−0.0180 (10)	0.0089 (10)
C13	0.0663 (14)	0.0285 (11)	0.0285 (10)	−0.0015 (10)	−0.0056 (10)	0.0038 (8)
C14	0.0653 (14)	0.0372 (12)	0.0418 (11)	−0.0217 (10)	−0.0031 (10)	0.0077 (9)
C15	0.0466 (12)	0.0392 (11)	0.0360 (10)	−0.0135 (9)	−0.0117 (9)	0.0058 (9)
C16	0.103 (2)	0.0465 (14)	0.0421 (12)	−0.0053 (13)	−0.0091 (12)	0.0139 (11)
C17	0.0561 (13)	0.0298 (11)	0.0351 (11)	−0.0083 (9)	−0.0089 (9)	0.0030 (9)
C18	0.0499 (12)	0.0307 (11)	0.0299 (10)	−0.0054 (9)	−0.0074 (9)	0.0039 (8)
N1	0.0766 (12)	0.0255 (9)	0.0401 (9)	−0.0073 (8)	−0.0172 (8)	0.0030 (7)
N2	0.0442 (9)	0.0267 (9)	0.0302 (8)	−0.0047 (7)	−0.0101 (7)	0.0042 (7)
N3	0.0529 (10)	0.0337 (9)	0.0257 (8)	−0.0053 (8)	−0.0105 (7)	0.0013 (7)
N4	0.1006 (16)	0.0303 (11)	0.0475 (11)	−0.0096 (10)	−0.0134 (10)	0.0014 (8)
N5	0.1054 (16)	0.0584 (13)	0.0359 (10)	−0.0146 (11)	−0.0165 (10)	−0.0068 (9)

### Geometric parameters (Å, °)

**Table:**

C1—N2	1.329 (2)	C7'—C8'	1.508 (16)
C1—N1	1.345 (2)	C7'—H7'1	0.9700
C1—C2	1.413 (2)	C7'—H7'2	0.9700
C2—C3	1.391 (2)	C8'—C9'	1.38 (2)
C2—C17	1.426 (2)	C8'—H8'1	0.9700
C3—C4	1.397 (2)	C8'—H8'2	0.9700
C3—C10	1.494 (2)	C9'—N3	1.453 (19)
C4—C18	1.426 (2)	C9'—H9'1	0.9700
C4—C5	1.436 (2)	C9'—H9'2	0.9700
C5—N3	1.343 (2)	C10—C11	1.382 (2)
C5—N2	1.350 (2)	C10—C15	1.386 (2)
C6—N3	1.457 (11)	C11—C12	1.377 (3)
C6—C7	1.523 (12)	C11—H11	0.9300
C6—H6A	0.9700	C12—C13	1.381 (3)
C6—H6B	0.9700	C12—H12	0.9300
C7—C8	1.515 (9)	C13—C14	1.380 (3)
C7—H7A	0.9700	C13—C16	1.503 (3)
C7—H7B	0.9700	C14—C15	1.382 (3)
C8—C9	1.518 (11)	C14—H14	0.9300
C8—H8A	0.9700	C15—H15	0.9300
C8—H8B	0.9700	C16—H16A	0.9600
C9—N3	1.486 (10)	C16—H16B	0.9600
C9—H9A	0.9700	C16—H16C	0.9600
C9—H9B	0.9700	C17—N5	1.144 (2)
C6'—N3	1.48 (2)	C18—N4	1.144 (2)
C6'—C7'	1.52 (3)	N1—H1A	0.8600
C6'—H6'1	0.9700	N1—H1B	0.8600
C6'—H6'2	0.9700		
			
N2—C1—N1	116.85 (15)	C9'—C8'—H8'1	110.7
N2—C1—C2	122.80 (15)	C7'—C8'—H8'1	110.7
N1—C1—C2	120.35 (15)	C9'—C8'—H8'2	110.7
C3—C2—C1	118.84 (15)	C7'—C8'—H8'2	110.7
C3—C2—C17	122.85 (16)	H8'1—C8'—H8'2	108.8
C1—C2—C17	118.29 (15)	C8'—C9'—N3	109.3 (14)
C2—C3—C4	118.92 (15)	C8'—C9'—H9'1	109.8
C2—C3—C10	119.08 (15)	N3—C9'—H9'1	109.8
C4—C3—C10	122.00 (15)	C8'—C9'—H9'2	109.8
C3—C4—C18	117.60 (15)	N3—C9'—H9'2	109.8
C3—C4—C5	118.67 (15)	H9'1—C9'—H9'2	108.3
C18—C4—C5	123.73 (15)	C11—C10—C15	118.23 (16)
N3—C5—N2	114.48 (14)	C11—C10—C3	120.58 (15)
N3—C5—C4	124.32 (15)	C15—C10—C3	121.11 (16)
N2—C5—C4	121.20 (15)	C12—C11—C10	120.90 (18)
N3—C6—C7	103.8 (7)	C12—C11—H11	119.6
N3—C6—H6A	111.0	C10—C11—H11	119.6
C7—C6—H6A	111.0	C11—C12—C13	121.73 (19)
N3—C6—H6B	111.0	C11—C12—H12	119.1
C7—C6—H6B	111.0	C13—C12—H12	119.1
H6A—C6—H6B	109.0	C14—C13—C12	116.77 (17)
C8—C7—C6	104.8 (5)	C14—C13—C16	121.75 (19)
C8—C7—H7A	110.8	C12—C13—C16	121.5 (2)
C6—C7—H7A	110.8	C13—C14—C15	122.44 (18)
C8—C7—H7B	110.8	C13—C14—H14	118.8
C6—C7—H7B	110.8	C15—C14—H14	118.8
H7A—C7—H7B	108.9	C14—C15—C10	119.89 (18)
C7—C8—C9	104.7 (4)	C14—C15—H15	120.1
C7—C8—H8A	110.8	C10—C15—H15	120.1
C9—C8—H8A	110.8	C13—C16—H16A	109.5
C7—C8—H8B	110.8	C13—C16—H16B	109.5
C9—C8—H8B	110.8	H16A—C16—H16B	109.5
H8A—C8—H8B	108.9	C13—C16—H16C	109.5
N3—C9—C8	102.4 (6)	H16A—C16—H16C	109.5
N3—C9—H9A	111.3	H16B—C16—H16C	109.5
C8—C9—H9A	111.3	N5—C17—C2	174.4 (2)
N3—C9—H9B	111.3	N4—C18—C4	177.49 (19)
C8—C9—H9B	111.3	C1—N1—H1A	120.0
H9A—C9—H9B	109.2	C1—N1—H1B	120.0
N3—C6'—C7'	103.1 (14)	H1A—N1—H1B	120.0
N3—C6'—H6'1	111.1	C1—N2—C5	119.50 (14)
C7'—C6'—H6'1	111.1	C5—N3—C9'	126.0 (8)
N3—C6'—H6'2	111.1	C5—N3—C6	127.7 (5)
C7'—C6'—H6'2	111.1	C9'—N3—C6	102.6 (8)
H6'1—C6'—H6'2	109.1	C5—N3—C6'	125.6 (9)
C8'—C7'—C6'	104.2 (11)	C9'—N3—C6'	108.3 (12)
C8'—C7'—H7'1	110.9	C6—N3—C6'	16.9 (8)
C6'—C7'—H7'1	110.9	C5—N3—C9	119.2 (4)
C8'—C7'—H7'2	110.9	C9'—N3—C9	15.9 (6)
C6'—C7'—H7'2	110.9	C6—N3—C9	112.7 (6)
H7'1—C7'—H7'2	108.9	C6'—N3—C9	114.2 (10)
C9'—C8'—C7'	105.2 (11)		
			
N2—C1—C2—C3	2.0 (3)	C16—C13—C14—C15	−177.78 (18)
N1—C1—C2—C3	−177.54 (16)	C13—C14—C15—C10	0.4 (3)
N2—C1—C2—C17	−179.85 (16)	C11—C10—C15—C14	−1.9 (3)
N1—C1—C2—C17	0.6 (3)	C3—C10—C15—C14	174.71 (17)
C1—C2—C3—C4	−2.3 (2)	N1—C1—N2—C5	179.88 (15)
C17—C2—C3—C4	179.63 (17)	C2—C1—N2—C5	0.3 (2)
C1—C2—C3—C10	177.00 (16)	N3—C5—N2—C1	177.57 (15)
C17—C2—C3—C10	−1.0 (3)	C4—C5—N2—C1	−2.2 (2)
C2—C3—C4—C18	−179.12 (16)	N2—C5—N3—C9'	−13.0 (7)
C10—C3—C4—C18	1.6 (2)	C4—C5—N3—C9'	166.8 (7)
C2—C3—C4—C5	0.5 (2)	N2—C5—N3—C6	−167.4 (4)
C10—C3—C4—C5	−178.81 (16)	C4—C5—N3—C6	12.4 (4)
C3—C4—C5—N3	−177.95 (16)	N2—C5—N3—C6'	171.6 (8)
C18—C4—C5—N3	1.7 (3)	C4—C5—N3—C6'	−8.6 (8)
C3—C4—C5—N2	1.8 (2)	N2—C5—N3—C9	4.0 (4)
C18—C4—C5—N2	−178.55 (16)	C4—C5—N3—C9	−176.2 (4)
N3—C6—C7—C8	24.5 (6)	C8'—C9'—N3—C5	−166.2 (7)
C6—C7—C8—C9	−34.4 (7)	C8'—C9'—N3—C6	−6.7 (12)
C7—C8—C9—N3	30.0 (6)	C8'—C9'—N3—C6'	9.8 (14)
N3—C6'—C7'—C8'	−25.3 (12)	C8'—C9'—N3—C9	124 (5)
C6'—C7'—C8'—C9'	32.1 (14)	C7—C6—N3—C5	166.3 (3)
C7'—C8'—C9'—N3	−26.1 (14)	C7—C6—N3—C9'	7.2 (8)
C2—C3—C10—C11	57.6 (2)	C7—C6—N3—C6'	−105 (5)
C4—C3—C10—C11	−123.05 (19)	C7—C6—N3—C9	−5.7 (6)
C2—C3—C10—C15	−118.87 (19)	C7'—C6'—N3—C5	−173.4 (6)
C4—C3—C10—C15	60.5 (2)	C7'—C6'—N3—C9'	10.5 (12)
C15—C10—C11—C12	1.5 (3)	C7'—C6'—N3—C6	83 (4)
C3—C10—C11—C12	−175.07 (17)	C7'—C6'—N3—C9	−5.3 (11)
C10—C11—C12—C13	0.3 (3)	C8—C9—N3—C5	172.1 (3)
C11—C12—C13—C14	−1.7 (3)	C8—C9—N3—C9'	−68 (4)
C11—C12—C13—C16	177.43 (19)	C8—C9—N3—C6	−15.2 (6)
C12—C13—C14—C15	1.4 (3)	C8—C9—N3—C6'	3.1 (8)

### Hydrogen-bond geometry (Å, °)

**Table:**

*D*—H···*A*	*D*—H	H···*A*	*D*···*A*	*D*—H···*A*
N1—H1A···N4^i^	0.86	2.19	3.010 (2)	160
C11—H11···N2^ii^	0.93	2.62	3.531 (2)	166

Symmetry codes: (i) *x*, *y*−1, *z*; (ii) −*x*, −*y*+1, −*z*+1.
